# Interactions with humans are jointly influenced by life history stage and social network factors and reduce group cohesion in moor macaques (*Macaca maura*)

**DOI:** 10.1038/s41598-019-56288-z

**Published:** 2019-12-27

**Authors:** Kristen S. Morrow, Hunter Glanz, Putu Oka Ngakan, Erin P. Riley

**Affiliations:** 1 San Diego State University, Department of Anthropology, San Diego, CA 92182 USA; 2 University of Georgia, Department of Anthropology and Integrative Conservation, Athens, GA 30602 USA; 3 California Polytechnic State University, Statistics Department, San Luis Obispo, CA 93407 USA; 4 Universitas Hasanuddin, Faculty of Forestry, Makassar, Sulawesi 90245 Indonesia

**Keywords:** Behavioural ecology, Animal behaviour

## Abstract

Human-wildlife encounters are becoming increasingly frequent across the globe, often leading people to interact with and feed wild animals and impacting animal behaviour and ecology. Although the nature of human-wildlife interactions has been well documented across a number of species, we still have limited understanding as to *why* some individual animals interact more frequently with humans than others. Additionally, we lack a comprehensive understanding of how these interactions influence animal social networks. Using behavioural data from a group of moor macaque monkeys (*Macaca maura*), we used permutation-based linear regression analyses to understand how life history and social network factors jointly explain interindividual variation in tendency to interact with humans along a provincial road in South Sulawesi, Indonesia. As our study group spent only a portion of their time in proximity to humans, we also examined how social network structure changes in response to human presence by comparing social networks in the forest to those along the road. We found that sex, individual network position, and associate network position interact in complex ways to influence individual behaviour. Individual variation in tendency to be along the road caused social networks to become less cohesive when in proximity to humans. This study demonstrates that nuanced intragroup analyses are necessary to fully understand and address conservation issues relating to human-wildlife interactions.

## Introduction

In an era characterized by rapid environmental change, wildlife populations are increasingly living in anthropogenic settings. These contexts can entail direct human influence, such as predation or provisioning, or indirect influence, such as habitat alteration or destruction. Conducting wildlife research in these explicitly anthropogenic settings is valuable in that scholars can explore contemporary evolution and expand our understanding of species’ adaptive potential^1^; such research is also necessary given that human influence on wildlife often exacerbates conservation concerns. Species at higher trophic levels with small ranges and slow life histories, such as primates, are especially likely to be negatively affected by frequent encounters with humans because they cannot rapidly recover from environmental disturbances^2^.

Changes to the environment or in human behaviour can alter previous patterns of human-wildlife coexistence and bring human and nonhuman animals in closer proximity to one another. Animals that consume anthropogenic food resources may display shifts in movement, behaviour, and ecology in response to human proximity, and humans may engage in provisioning behaviour in response to animal presence^3–7^. Food provisioning, whereby humans purposely give food to nonhuman animals, can result in rapid habituation of wildlife such that they begin to approach humans for food, take food from their hands, and potentially aggress toward them to elicit provisioning behavior^3,7–9^.

For primates, because provisioning can result in decreased foraging energy expenditure, increased time available for resting and social behaviour, and increased birth rates^10–12^, provisioned foods can be valuable energetic resources. Nevertheless, provisioning can also be extremely risky for primates and other wildlife species as it is associated with increased risk of direct conflict with people, zoonotic disease transmission, and injury^13–16^. Previous research indicates that life history stage is an important factor shaping individual animals’ willingness to interact with people or engage with anthropogenic habitat features, with males, especially older males, interacting more frequently with humans than females, and juveniles’ interactions with humans varying across species^15,17–19^.

Life history theory predicts that differential fitness expectations and energy requirements will cause age-sex classes to vary in risk-taking behavior^20,21^. In anthropogenic contexts, such variation may be visible via intraspecific differences in willingness to interact with humans to exploit human food resources^17^. In primates, adult and subadult males are expected to be risk-prone due to high current reproductive potential, need to compete for mates, and lower mortality risk, whereas adult females are expected to be risk averse due to high cost of reproduction; juveniles are expected to be somewhat risk-averse due to higher mortality risks and low current reproductive potential^20,22,23^. By examining variation in behaviour across age-sex class and by examining patterns of risk response—such as increased vigilance behaviour or decreased interindividual distance^24^—life history theory predictions can be tested.

However, when considering species with complex social groups, the relationships and interactions with group members must also be considered when attempting to understand behaviour. For instance, association patterns within a social group can influence group movement patterns^25–28^, individuals’ risk-taking behaviour^17,24^, and group cohesion and stability^12,29^. Social network analysis (SNA) offers a powerful tool to investigate animal sociality and explore how changes in environmental and social context shape group-wide patterns^30–32^. Using social network analysis to examine the causes and effects of human-wildlife interaction represents a promising application of this tool that has yet to be explored fully^17^^,^^33,34^. Although there is a growing number of studies examining how behaviour, life history stage, and context all interact to influence network position^35,36^, we know of no other studies examining the inverse—how life history stage and network position influence individual behavioural patterns.

In this study, we examine how intrinsic life history factors and extrinsic social network factors jointly influence how often moor macaque (*Macaca maura*) monkeys are in proximity to provisioning opportunities along a heavily trafficked road that bisects Bantimurung Bulusaraung National Park (BBNP) in South Sulawesi, Indonesia. The moor macaque (*Macaca maura*) is an Endangered primate species endemic to South Sulawesi, Indonesia^37^. While people have long encountered moor macaques during crop foraging events at the forest-farm edge throughout the species’ distribution^38^, frequent, direct interactions with wild primates in BBNP were uncommon until recently. Beginning in 2015, our macaque study group shifted their behaviour, spending more time along the provincial road bisecting its home range, where some individuals began approaching passing vehicles and accepting food from humans. This group has been habituated to human presence for many years due to occasional provisioning by BBNP staff and regular following by researchers, but had previously not spent time along this road. Because our study group still spends a large portion of their time in forested habitat, away from provisioning opportunities found along the road, we were able to test life history predictions while also exploring how “typical” patterns of social interaction (i.e., behaviour in the forest) influence macaque tendency to be along the road. This context also allowed us to assess the effects of interacting with humans by comparing social networks along the road to social networks in the forest.

From a life history perspective, we expected that adult and subadult males would be the most risk-prone, and thus be along the road in proximity to humans most often, followed by juveniles then adult females. We expected that the macaques would perceive the road to be risky, and that this perception would be evidenced by increased proximity to group members while along the road. From a social network perspective, we explored how commonly used measures of centrality interact with life history factors to explain individual tendency to be in proximity to the road. Given that we expected intragroup variation in time spent near the road, we expected that proximity patterns observed in the forest context would be disrupted while the group was along the road. Additionally, although predictable, consistent provisioning or human presence may increase frequency of social behaviour^39^, inconsistent provisioning—e.g., provisioning performed from moving vehicles and hence not spatially concentrated—can disrupt social behaviour^39,40^. Thus, we predicted that proximity-based and affiliative-based social networks along the road would be less cohesive and more fragmented than social networks within the forest.

## Methods

### Study site and subjects

**Figure 1:**
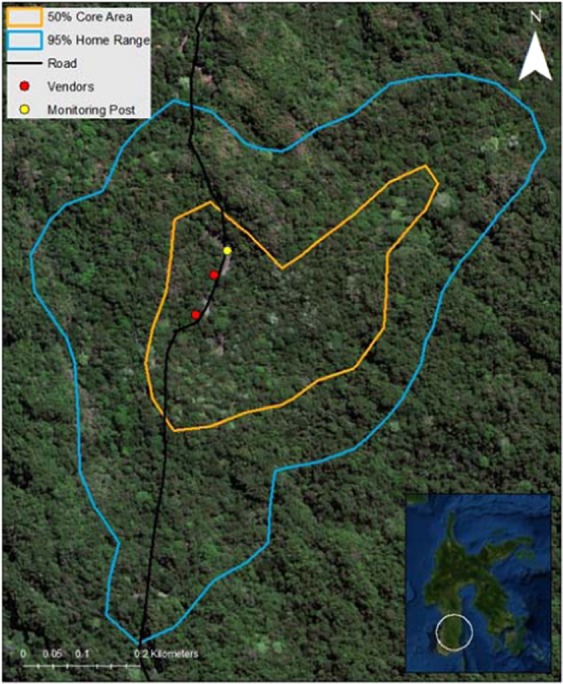
Map of study site in Sulawesi, Indonesia illustrating the location of the road and other anthropogenic features (e.g., vendor stalls and a monitoring post) in relation to the study group’s home range and core area. Imagery source: DigitalGlobe (2014). Map created using the ArcGIS software version 10.7.1 by Esri: http://desktop.arcgis.com/en/arcmap/.

We observed a habituated group of moor macaques (*Macaca maura*) in the Karaenta area of Bantimurung Bulusaurang National Park in South Sulawesi, Indonesia (Fig. 1). Established in 2004, BBNP protects approximately 43,000 ha of the region’s karst (limestone) ecosystem and biodiverse flora and fauna, including the Endangered moor macaque. Karaenta is a 1000-ha section of BBNP characterized by mixed primary and secondary forest and seasonality in rainfall (typically, wet season: November – May; dry season: June – October). Although the northern portion of Karaenta borders residential and agricultural areas, our study group’s range does not as it is approximately 3 km from the nearest village. Aside from research, limited ecotourism, and small-scale, non-timber resource extraction, Karaenta is not used by humans. However, roadside vendors sell forest goods (e.g., honey) along the road bisecting the park, and some vendor stalls can be found within our study group’s home range. Additionally, a park-constructed monitoring post within our study group’s home range was frequently used as a resting and eating area by passing motorists (Fig. 1). Private vehicles, public transportation vehicles, and large trucks transporting commercial goods heavily traffic the road that bisects Karaenta. Food waste and larger trash items (e.g., construction materials) are frequently left along the road, and people are increasingly inclined to stop, rest, eat, or purchase products sold by roadside vendors.

**Figure 2:**
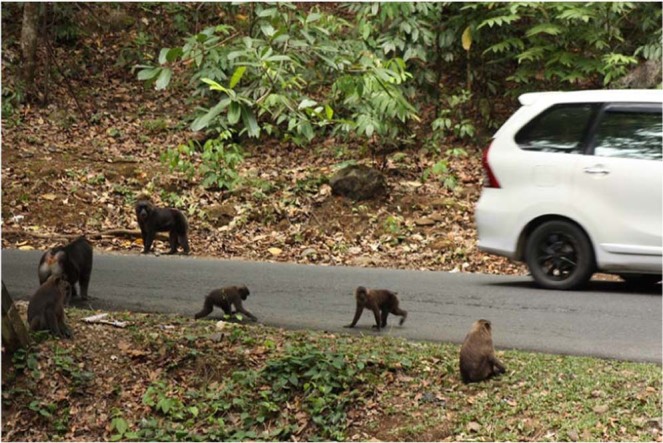
Vehicles stop along road to provision wild moor macaques (*Macaca maura*) in South Sulawesi, Indonesia. Photo credit: Kristen S. Morrow.

A number of *M. maura* groups inhabiting Karaenta have home ranges that encompass both the north and south side of the road and are frequently seen crossing the road as they travel through their home range. This road-crossing behaviour (Fig. 2) creates the opportunity for people to view, photograph, and provision wild macaques. Although provisioning itself consistently occurred when our study group was near the road within their home range (Fig. 1), the exact location of provisioning, quantity of food provisioned, and number of people provisioning simultaneously was inconsistent. The macaques frequently rested along the road while monitoring passing vehicles. Provisioned foods, which typically included cultivated fruit, bread, and chips, were most commonly tossed from inside stopped or slowly moving vehicles, though people would occasionally get out or off of stopped vehicles and approach the macaques. We often observed the macaques following vehicles that slowed in their proximity. Although we witnessed no injuries to macaques from vehicles during the study period, vigilance behaviour while crossing the road was regularly observed and several individuals have since been observed being hit and killed by passing vehicles along the road.

Moor macaques live in cohesive multimale, multifemale groups, display female philopatry, and frequently display behavioural synchrony—i.e., engage in the same behaviour in time and space^11,41^. *M. maura* is a socially tolerant species, characterized by symmetry in aggression, high rates of affiliation and reconciliation, low influence of kinship on social interactions, tolerance of feeding near non-kin, and tension-reducing behaviours during male-male interactions^42,43^. Humans and larger snakes are the only potential predators of this species. Although there is no evidence that people in the area hunt *M. maura* for consumption, there are reports of retaliation against crop feeding macaques across the species’ range^38,44^. Our study group comprised thirty-five individuals during the period of data collection: nine adult males (≥8 years old), 11 adult females (>6 years old), two subadult males (6–7 years old), six juvenile males (1–5 years old), five juvenile females (1–4 years old), and two infants (<1 year old). All individuals were recognizable based on natural markings. Age classes were estimated according to body size and appearance of external genitalia^11,43,45^. Individuals that were not present for the entirety of data collection (n = 1 adult male), as well as infants (n = 2), were excluded from data analysis.

### Data collection

Data were collected six hours per day, six days per week from August 2016 to January 2017, rotating morning (0600–1200) and afternoon (1200–1800) sampling periods. Data collection began during the dry season, which lasted until November 2016. We collected macaque behavioural data using scan sampling^46^ every 30 minutes, with each scan lasting for 10 minutes. During each scan sample, we attempted to record each individual’s location (i.e., along the road or in the forest), identity of all individuals within 1 m, and behavioural state. Location was scored as “along the road” if the individual was on the road or in roadside areas that lacked forest vegetation; all other locations were scored as “in the forest.” We chose a low proximity threshold of 1 m to record nearest neighbours because socially tolerant species often display low interindividual distances^47^. Behavioural states included affiliative behaviour, aggressive behaviour, grooming (allo- and auto-), feeding, foraging, following conspecifics, locomote, male-male greetings, rest, sexual behaviour, and play behavior^43,48^.

### Building comparable social networks

Because our study group ranges in areas with and without human presence, it presented a unique opportunity to examine how proximity to provisioning opportunities impact macaque social networks. Because networks can vary based on data included^49–51^, we constructed and examined two network types in two contexts: networks based on affiliative interactions and networks based on proximity patterns, in both forest and road contexts.

When comparing animal social networks, it is ideal to have the same individuals in the compared networks, to sample equally across comparison contexts, and to construct networks using data collected during the same temporal period^50–52^. In studies relying on observational data from wild animals, these requirements often cannot be met. Although our networks comprise the same individuals and are constructed from data collected during the same temporal period, we have an unequal number of observations across individuals and our study group spent much more time in the forest than along the road. To address the first concern, we weighted each network with association indices^53,54^. To address the bias in sampling across road and forest contexts, we compared a comprehensive forest network using all scans in the forest to an estimated forest network^55,56^. The estimated forest network was constructed by generating 100 random samples of scans in the forest. Each sample contained the same number of scans observed along the road and followed the same monthly pattern as scans along the road (e.g., 42 scans were recorded along the road in October and 42 forest scans recorded in October were included in each random sample). For each random sample we calculated half-weight association indices for all pairs of individuals. We then averaged each dyad’s half-weight association index values across all 100 samples and used these averaged values to construct the estimated network. We used Spearman’s rank correlation analyses to assess whether network metrics were similar across comprehensive and estimated forest networks^55^.

Across road and forest contexts a total of six social networks were constructed: a road affiliative network, a road proximity network, a comprehensive forest proximity network, a comprehensive forest affiliative network, an estimated forest proximity network, and an estimated forest affiliative network.

#### Association indices

We created half-weight association indices (HWI) for all possible pairs of individuals (n = 992 possible pairs) in our study group. The HWI index provides a coefficient for the proportion of observations a pair of individuals was observed together, accounting for biases that arise from having a different number of total observations per individual^53^. Weighting social networks with HWI values, instead of frequency of interactions or joint observations, allows for the construction of multiple social networks that can be compared despite differences in number of observations per network^50,51,54^. HWI is calculated as *2 N/(n*_*A*_ + *n*_*B*_*)*, where *2 N* is the total number of joint sightings (i.e., dyadic interactions or proximity within 1 m) for individuals *A* and *B*, *n*_*A*_ is the total number of sightings of individual *A*, and *n*_*B*_ is the total number of sightings for individual *B*. Reciprocal joint sightings (i.e., individual A interacting with B, and vice versa) within a single scan were counted only once.

We calculated different sets of HWI values for affiliative interactions and proximity-based associations in the forest and along the road. For each set of HWI values, the number of joint sightings *N* and the number of individual sightings *n*_*A*_ and *n*_*B*_ were both calculated within context (road/forest). For example, when calculating the forest affiliative HWI for individuals *A* and *B, N* represents all affiliative interactions between *A* and *B* that occurred in the forest, *n*_*A*_ represents the total number of sightings of individual *A* in the forest, and *n*_*B*_ represents the total number of sightings of individual *B* in the forest. As proximity-based analyses were non-directed, HWI values in proximity-based subsets were reciprocal; i.e., the HWI value for dyad *A-*B matched that of dyad *B-*A.

### Data analysis

#### Assessment of risk perception

To assess whether age-sex classes were in closer proximity to group members while along the road (i.e., to assess risk perception), we compared by age-sex class the average number of conspecifics within 1 m across road and forest contexts using the Mann-Whitney U tests evaluated at α = 0.05.

#### Understanding tendency to interact with humans

Similar to previous research^33^, we used modified, permutation-based linear regression models to understand how life history and typical social dynamics jointly explain individual macaque proportion of behavioural records along the road. Predictor variables included individual age (adult or nonadult), sex (male or female), eigenvector centrality, betweenness centrality, top associate eigenvector centrality, and top associate betweenness centrality. Social network variables were derived from the comprehensive proximity-based forest network; each individual’s network metric values represent their network position throughout the entire study period. Top associates were identified by determining each individual’s strongest relationship in the group, as measured by the greatest HWI value. Interaction terms reduced model explanatory value and were not included. We selected variables and interaction terms based on assessments of biological relevance and selected models based on overall explanatory value. Although network measures are relative to one another, no network variables in our model were strongly correlated with other included network metrics.

In the permutation-based linear regression model approach, estimated regression coefficients indicate the extent to which an individual’s proportion of behavioural records along the road is expected to change in response to a change in a given predictor variable. Using the lmPerm package in R^57^, we permuted the values of the response variable, maintaining the value of the predictor variables, until the estimated p-values converged, or until the maximum iterations (n = 5000) had been completed. In the lmPerm package approach, the structure in the covariates is maintained as the null distribution of the covariates are generated via permutation of the response. Estimated two-tailed p-values were assessed at α = 0.05.

Although dominance rank is a commonly investigated factor in studies of primate social relationships and has been included in previous social network studies and studies on human-macaque interactions^33,58^, we had insufficient data on agonistic interactions to reliably construct individual dominance rank to include as a variable in our analyses. However, moor macaques have more socially tolerant relationships, tolerate proximity to one another while feeding, and likely experience low within-group competition^42,43,59^. These traits, combined with the spatially dispersed nature of provisioning along the road, likely reduces the influence of dominance rank on social interactions and access to provisioned foods. As such, we suggest that excluding dominance rank does not prevent meaningful interpretation of our analyses.

Two network metrics used in comparison of networks across context (Table 1) were not included in permutation-based linear regression models: closeness centrality and degree centrality. Closeness centrality was excluded both to simplify model complexity and to facilitate comparison with related studies^33^. Degree centrality was not included because it was highly correlated with eigenvector centrality in proximity-based forest networks (Spearman’s r_s_ = 0.983, p < 0.001); given this high correlation, we removed degree centrality from regression analyses. Although both metrics are frequently used in network studies, eigenvector centrality represents a more robust measurement of centrality within a group as it takes into consideration both direct and indirect connections^60^ and is increasingly found to be an important metrics in primate social networks^28,61^.

**Table 1:** Social network metrics used for comparison of road and forest metrics and for regression analyses.

Metric	Definition & Usage
Density^84^ (global measure)	The proportion of all possible edges that are present in a network. Used to assess group connectedness.
Weighted Degree Centrality^84^	The sum of the weights of the edges connected to an actor *i*. Used to assess “importance” of an individual within a group based on all their ties to other individuals.
Betweenness^84^	The sum of the edge weights from the geodesic (shortest) paths connecting two nodes that pass through an actor *i*. Used to assess the “importance” of an individual based on their role in connecting other pairs of individuals.
Closeness^84^	The inverse of the sum of the geodesic distances from actor *i* to all the other actors in a network. Used to assess the “importance” of an individual based on how their position within a network allows them to quickly interact with other individuals.
Eigenvector Centrality^60^	The composite centrality scored based on principal eigenvector values provided by the adjacency matrix of a graph; this measure takes into account both a node’s and that node’s connections’ eigenvalues. Used to assess an individual’s “importance” in the network based on their own centrality and the centrality of their network connections.

For regression analyses we separated the age (i.e., adult, non-adult) and sex (i.e., male, female) variables in order to assess the different ways each of these life history traits influence individual behaviour, and to maintain a larger sample size in each category. Most individuals in our study group are easily classified as either adults or non-adults, with the exception of subadults (n = 2 males, 0 females). For the purposes of regression analyses, we coded subadult individuals as adults. In many primate species males disperse at sexual maturity, and the subadult period represents a stage of increased energetic need and less social interactions with group members^62,63^. Both subadult males in our study group were close to adult size and were observed engaging in copulation with adult females on multiple occasions; as such, we concluded their energetic needs and behaviour more closely matched that of adults and that they could be considered as such.

#### Assessing impact of proximity to humans

We assess the impact that proximity to humans has on macaques by comparing social networks across road and forest contexts. Affiliative interaction networks were directed, whereas proximity-based networks were undirected. The inclusion of both interaction-based and proximity-based networks allowed us to assess the methodological implications of data type on network analyses; as such, we report both here. For all networks, edge weights were defined by the HWI values specific to the context and data type. Network metrics and visualizations were calculated in R (version 3.3.1) using the statnet^64^, network^65^, sna^66^, igraph^67^, and RColorBrewer packages^68^. Table 1 provides a list of and details on the metrics compared across context, which were selected based on their prevalence in the literature and ability to assess different aspects of social relationships.

To assess whether metrics in the comprehensive forest network differed from those in the estimated forest network, we used Spearman’s rank correlation analyses^55^. We found that metrics were not consistently correlated across comprehensive and estimated forest networks. In particular, closeness centrality was not significantly correlated in proximity forest networks (r_s_ = −0.80, p = 0.13), and eigenvector centrality was not significantly correlated in either proximity (r_s_ = 0.80, p = 0.13) or affiliative networks (r_s_ = −0.01, p = 0.97). We therefore compared the estimated forest network to the road network in order to assess how proximity to humans influenced our study group.

To examine how metrics in forest networks differed from metrics in road networks, we used randomization tests, a common approach to compare observed and simulated social networks^32,34,69^. For both interaction and proximity-based networks, and for each network metric analysed, we first subtracted each individuals’ metric value in the road network from their metric value in the estimated forest network. This resulted in eight sets of differences (one per network metric per data type). Then, 10,000 simulated sets of differences were generated by sampling from the observed differences. The mean difference for each of the 10,000 generated sets were compared to the observed mean difference for each metric. Approximate two-tailed p-values evaluated at α = 0.05 were calculated for each comparison. As randomization tests were used, no assumptions of normality distributions needed to be met. Randomization-based comparisons of network metrics were calculated in the stats package^70^ of R (version 3.31.1).

We also descriptively assessed changes in network metrics across context by age-sex class and assessed differences in network clustering across context. For the latter, we used the walk-trap community algorithm, a recommended clustering algorithm for social networks with <1000 nodes^71^. This method detects clusters of densely connected individuals by using random walks; a modularity score, *Q*, is calculated for each subgroup detected. The network is then partitioned into the clusters which allow for maximum *Q*^69,71^. With this method, each detected cluster will have stronger ties within their cluster than between clusters.

### Ethical approval

Research permits were obtained from RISTEK-DIKTI (Permit # 212/SIP/FRP/E5/Dit.KI/VIII/2016) following Indonesian research protocol. All methods were approved by the Institutional Animal Care and Use Committee at San Diego State University (APF # 14-03-006R).

## Results

Across the study period, we followed the study group for 565.8 hours, completing a total of 1,219 scan samples (1,170 in contact with the group, 195 hours). The study group was along the road for 19.8% of all scan samples (n = 232 scans). Although the group was located along the road at various times throughout the day, the group was most often observed along the road between 11:00–17:00 hours (68.9% of scan samples along the road, n = 159). The group was infrequently along the road before 0700 or after 1700. Averaging across all scans, subadult males were observed in proximity to the road in the largest percentage of behavioural records (26.7% ± s.d. 1.4%) followed by adult males (19.9% ± s.d. 6.7%), juvenile males (18.4% ± s.d. 2.5%), juvenile females (14.5% ± s.d. 3.8%), and adult females (12.1% ± s.d. 3.6%).

### Assessment of risk perception

All age-sex classes had, on average, fewer nearest neighbours per behavioural record while along the road as compared to while in the forest. This difference was significant for adult males (forest mean ± s.d. = 0.54 ± 1.03 vs. road mean ± s.d. = 0.39 ± 0.70, p = 0.002) and subadult males (forest mean ± s.d. = 0.53 ± 0.92 vs. road mean ± s.d. = 0.38 ± 0.67, p = 0.004). Differences in average number of nearest neighbours was not significantly different across road and forest contexts for adult females (forest mean ± s.d. = 0.74 ± 1.19 vs. road mean ± s.d. = 0.71 ± 0.98), p = 0.26, juvenile males (forest mean ± s.d. = 0.62 ± 1.01 vs. road mean ± s.d. = 0.52 ± 0.83, p = 0.06), or juvenile females (forest mean ± s.d. = 0.84 ± 1.23 vs. road mean ± s.d. = 0.75 ± 1.07, p = 0.48). Across road and forest contexts, adult females and juvenile females had, on average, the greatest number of nearest neighbours per behavioural observation compared to other age-sex classes.

### Factors influencing tendency to interact with humans

Based on data derived from the comprehensive forest proximity network, sex, betweenness centrality, and top associate eigenvector centrality all have a significant effect on individual proportion of behavioural records along the road (Table 2). Regardless of age, males were, on average, along the road significantly more often than females (p = 0.006). Individuals that are more central in the social network (as measured by increasing betweenness centrality) were along the road more often (p = 0.038). However, associate eigenvector centrality was a stronger predictor of the proportion of behavioural records along the road than individual betweenness centrality, such that individuals associated with more central group members (as measured by increasing associate eigenvector centrality) were along the road less often (p = 0.03). Age, individual eigenvector centrality, and associate betweenness centrality did not have a significant influence on individual proportion of behavioural records along the road (Table 2).

**Table 2:** Results of permutation-based linear regression model, evaluated at alpha of 0.05.

Variable	Coefficient	Iterations	p-value
Intercept	13.60	885	0.102
Age	0.18	51	0.922
Sex	5.65	5000	0.006*
Eigenvector Centrality	−0.65	51	0.843
Betweenness Centrality	0.07	2543	0.038*
Associate Eigenvector Centrality	−15.00	3279	0.030*
Associate Betweenness Centrality	−0.027	61	0.623

Network metrics and associate data derived from proximity-forest network. P-values denoted with * are significant. Adjusted R^2^ = 0.521, p < 0.001. For age, the comparison group is adults and the intercept represents non-adults; for sex, the comparison group is male and the intercept represents females. Maximum iterations of permutations of null models = 5,000.

### Proximity to humans disrupts social networks

**Figure 3:**
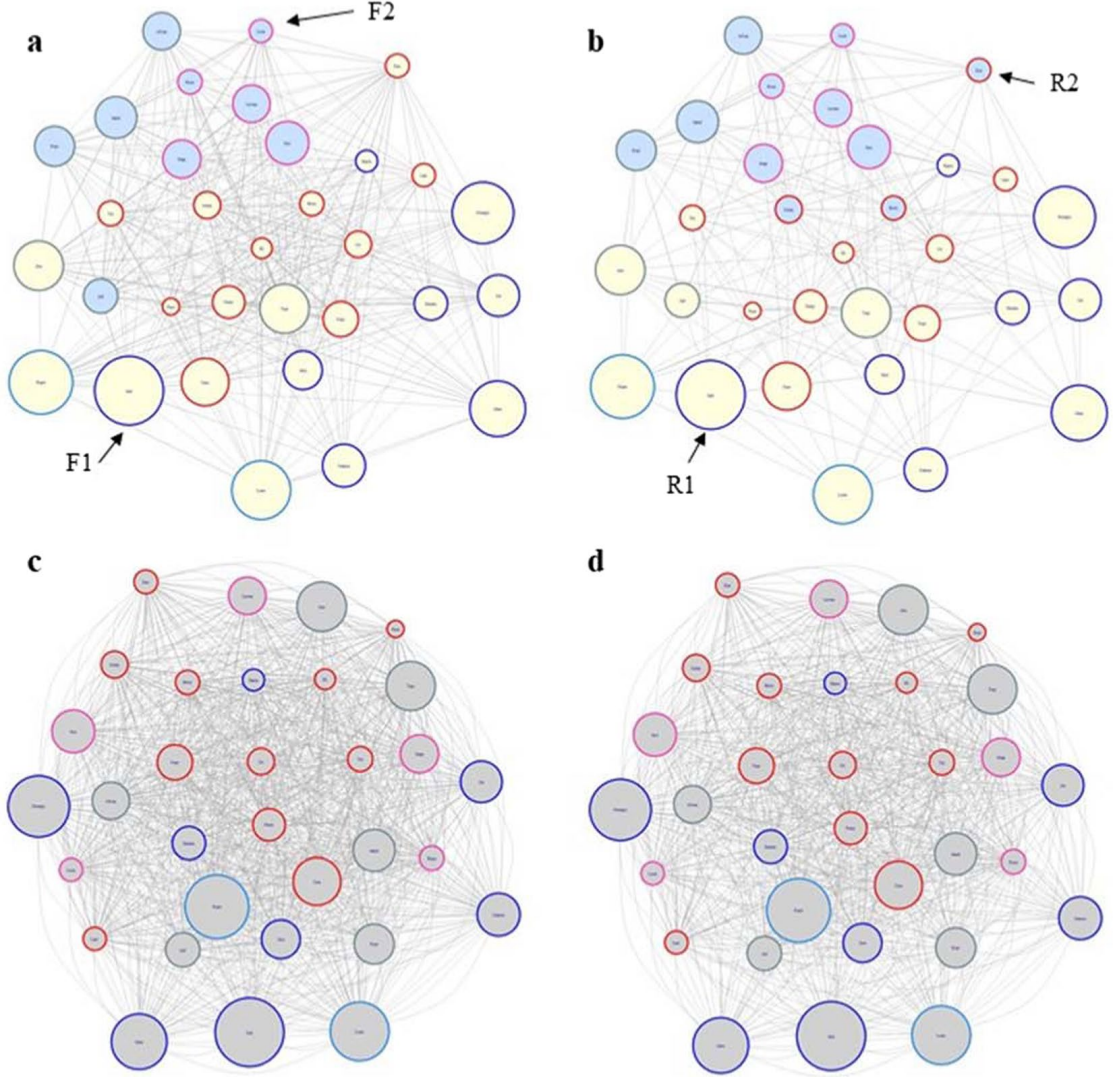
Affiliative forest (**a**) and road networks (**b**) and proximity forest (**c**) and road (**d**) networks. Across all networks, larger node size indicates greater proportion of behavioural records along the road. Node outline colour indicates age-sex class: adult males are outlined in blue (blue), adult females are outlined in red (red), subadult males are outlined light blue (dogerblue2), juvenile males are outlined in turquoise (lightblue4), and juvenile females are outlined in pink (maroon1). Node fill colour indicates membership in clusters identified via the walktrap community algorithm; text and arrows indicate cluster identities.

Regardless of data type, forest networks were denser than road networks (forest affiliative network 0.511 vs. road affiliative network: 0.280; forest proximity network: 0.957 vs. road proximity network: 0.843, Fig. 3). On average, weighted degree centrality was greater along the road and differed significantly more than randomized differences for both network types (affiliative: p < 0.001; proximity: p < 0.001). Conversely, betweenness centrality and closeness centrality were both greater in the forest than along the road. Closeness centrality differed significantly more than randomized differences across both network types (affiliative network: p < 0.001; proximity network: p < 0.001), whereas betweenness centrality only differed more than randomized differences in the proximity network (p = 0.03). Eigenvector centrality differences were not significantly different than randomized differences across either affiliative or proximity networks (Table 3).

**Table 3:** Comparison of differences between road and forest network metrics.

Network Type	Context with greater metric value	Observed mean difference ± s.d.	Average estimated mean difference ± s.d.	Estimated p-value
**Affiliative**				
Degree centrality	Road	0.12 ± 0.06	0.000 ± 0.01	<0.001*
Betweenness	Forest	−11.66 ± 60.53	0.10 ± 10.52	0.27
Closeness	Forest	−22.78 ± 6.89	0.004 ± 1.20	<0.001*
Eigenvector centrality	Forest	−0.04 ± 0.39	0.001 ± 0.07	0.52
**Proximity**				
Degree centrality	Road	0.78 ± 0.20	0.000 ± 0.04	<0.001*
Betweenness	Forest	−8.97 ± 27.42	0.02 ± 4.79	0.03*
Closeness	Forest	−6.71 ± 1.73	0.000 ± 0.30	<0.001*
Eigenvector centrality	Road	0.05 ± 0.24	0.000 ± 0.04	0.25

P-values denoted with * are significant at α = 0.05.

Clustering patterns vary across network type and context. No clusters were detected in the proximity networks (forest proximity network: *Q *= 0; road proximity network: *Q *= 0). Two clusters were identified in each of the affiliative networks (forest affiliative network: *Q* = 0.096; road affiliative network: *Q* = 0.15), with the greater modularity score in the road affiliative network indicating a less cohesive network along the road as compared to in the forest. The forest affiliative network was largely clustered based on age; cluster F1 (n = 23 individuals) contains all adult individuals and two juvenile males, whereas cluster F2 (n = 9 individuals) contains all other juveniles. Cluster composition in the road affiliative network was more varied; cluster R1 (n = 21 individuals) contains all adult and subadult males (n = 10 individuals), five adult females, and three juvenile males; cluster R2 (n = 11 individuals) contains three adult females, three juvenile males, and all juvenile females (n = 5 individuals) (Fig. 3).

Based on descriptive statistics, there was marked variation across age-sex class in mean difference of network measures. Adult females and juvenile females show the greatest increase in weighted degree centrality along the road across both network types, indicating more frequent affiliative interactions and proximity to group members. Conversely, subadult males showed the least increase in weighted degree centrality along the road, which indicates less pronounced changes in behaviour along the road compared to the forest. Adult females and juvenile females also show the greatest differences in affiliative betweenness centrality—indicating a change in their role indirectly connecting other group members to one another. However, whereas adult females play a lesser role connecting group members while along the road, juvenile females play a greater role connecting group members while along the road as compared to the forest. Juvenile males are the only other age-sex class that display increased betweenness centrality while along the road. In proximity networks, adult males and subadult males display the greatest decline in betweenness centrality across contexts, indicating their role connecting other group members changes the most due to proximity to humans. Adult males and adult females show the greatest decline in closeness centrality in affiliative networks, indicating that while along the road they are less connected to other group members than when in the forest. Juvenile females show the least change in closeness centrality across both affiliative and proximity networks, indicating that—compared to other age-sex classes—they maintain more similar social patterns across road and forest contexts. Adult males are the only age-sex class to show an increase in affiliative eigenvector centrality along the road (i.e., become more central in terms of their own interactions and their associates’ interactions), whereas juvenile females are the only age-sex class to show a decrease in proximity eigenvector centrality along the road (Table 4).

**Table 4:** Mean differences in social network metric across context, by network type and age-sex class.

Network Type	Degree Centrality ± s.d.	Betweenness ± s.d.	Closeness ± s.d.	Eigenvector Centrality ± s.d.
**Affiliative**				
Adult male	0.09 ± 0.04	−10.00 ± 40.54	−24.89 ± 6.13	0.25 ± 0.46
Adult female	0.13 ± 0.05	−48.27 ± 80.08	−26.43 ± 5.61	−0.10 ± 0.29
Subadult male	0.06 ± 0.00	−20.00 ± 14.14	−21.94 ± 9.34	−0.42 ± 0.33
Juvenile male	0.08 ± 0.03	21.83 ± 25.89	−18.76 ± 4.94	−0.10 ± 0.29
Juvenile female	0.20 ± 0.08	30.80 ± 31.08	−14.94 ± 3.77	−0.18 ± 0.44
**Proximity**				
Adult male	0.73 ± 0.27	−18.25 ± 41.37	−7.71 ± 2.14	0.13 ± 0.28
Adult female	0.86 ± 0.20	−2.00 ± 12.50	−6.52 ± 1.09	0.11 ± 0.14
Subadult male	0.68 ± 0.02	−25.00 ± 65.05	−7.61 ± 2.47	0.12 ± 0.22
Juvenile male	0.71 ± 0.15	−0.67 ± 12.19	−6.75 ± 1.85	0.02 ± 0.29
Juvenile female	0.81 ± 0.15	−13.00 ± 25.33	−5.09 ± 0.78	−0.21 ± 0.16

## Discussion

Overall, our study group spent around one-fifth of the observation period in proximity to the road, and many individuals actively engaged with people (e.g., approaching stopped vehicles). However, contrary to our expectations and previous research^24^, the macaques did not decrease interindividual distance while along the road, indicating they may not perceive the road context to be risky. These results suggest that the macaques are not merely habituated to human presence, but rather, are attracted to humans due to the food rewards obtained from provisioning^6,72^. Alternatively, this pattern may reflect that provisioning events occur simultaneously in multiple locations, thus potentially leading the macaques to be more dispersed while along the road despite perceived risks.

Regression analyses, on the other hand, support our predictions of life history-based risk aversion. Even when considering multiple life history and social network factors, males are more likely to be along the road than females. These findings lend further support to life history theory’s prediction that males will be more risk-prone^20,21^, and align with findings from past studies across multiple primate species^12,15,19,73^. The finding that non-adults were along the road somewhat often contradicts life history theory expectations^22,23^, but has been observed in other primate species^12^. Our findings underscore the challenges in identifying behavioral responses to risk in animals. While we find that sex class influences risk-taking behavior along the road, age-sex class patterns in average number of nearest neighbors across road and forest contexts does not clearly indicate life history-based differences in risk-taking. Additional data on behaviour while along the road, such as road crossing order, waiting time before crossing, or vigilance behaviour while crossing would further illuminate risk perception and responses to perceived risk^73,74^.

Similar to prior research^33^, network metrics also affected individual proportion of behavioural records in proximity to the road. However, our results demonstrate that different measures of network centrality differentially explain individual tendency to be in proximity to humans. Individuals that play a greater role in connecting other group members (i.e., have higher betweenness centrality) are more likely to be along the road. Conversely, individuals associated with highly integrated individuals (i.e., associates that have higher eigenvector centrality) are less likely to be along the road. Thus, when considering betweenness centrality, greater integration increases the likelihood of risk taking, whereas when considering associate eigenvector centrality, greater integration decreases likelihood of risk taking. Enhanced social network integration makes collective movement more efficient^28^ and improves likelihood of successful immigration^61^; these recent insights and our findings suggest that integration into social networks may be one mechanism through which stronger social bonds provide primates fitness benefits^75–77^. Notably, however, in our data, this relationship depends on the network metric being considered, highlighting the need to consider multiple network measures when investigating the relationship between network position and individual traits.

We found support for our prediction that social networks along the road would be less cohesive than social networks within the forest. Regardless of network type, social networks had lower density— i.e., were less cohesive^32,78^—along the road than in the forest. The loss of cohesion is further demonstrated by the greater cluster modularity score *Q* in the road affiliative network compared to the forest affiliative network. The lack of clusters detected in either forest or road proximity networks matches the expectation that socially tolerant macaques typically distribute social contacts evenly across a group^42,78,79^. Additionally, measures of inter-individual connections (i.e., closeness and betweenness centrality) were lower along the road than in the forest for both affiliative and proximity networks, indicating that typical connections between individuals were disrupted when in proximity to humans.

Previous experimental research shows that network centrality may increase in provisioning contexts^34^. Our results partially support this finding; in both affiliative and proximity networks, weighted degree centrality was greater along the road than in the forest. In other words, the relative frequency of affiliative interactions and close proximity to group members increased when our study group was along the road. However, we also found that all age-sex classes had, on average, fewer nearest neighbours while along the road as compared to when in the forest. Together, these results demonstrate that while provisioning may increase the relative frequency with which individuals interact with or are close to one another, it simultaneously disrupts the typical distribution of social interactions among group members such that individuals do not associate with the same diversity of group members along the road as they do in the forest.

The observed differences between road and forest networks supports the idea that primates can flexibly adjust network structure in response to environmental and social changes^31,80^ while providing additional evidence to a growing body of research demonstrating that interactions with humans disrupt primate social processes^34,40^. Indeed, the differences in road and forest networks found here highlights how severely proximity to humans disrupts macaque social behaviour, as patterns of macaque social relationships are typically highly resilient and stable across different contexts^28,78,79^. In the short term, such loss of cohesion can lead to increased aggression^80^. Over time, this disruption could compromise some of the many benefits primates derive from group living, including decreased predation risk, increased foraging efficiency and resource protection, and increased survival rates^75,81,82^. However, the finding that metrics did not uniformly differ across affiliative- and proximity-based networks points to the methodological importance of considering data type and metrics included in network analyses. Although some scholars^49^ argue that proximity-based networks are insufficient for understanding animal social relationships, we argue that examining both affiliative and proximity networks enhances our understanding of anthropogenic effects on social networks.

Descriptive statistics of social network metrics at the level of age-sex class are similarly useful, though more rigorous statistical testing with larger sample sizes would be beneficial. Our data show that being in proximity to the road results in some age-sex classes becoming more disconnected from the group than others, but these outcomes vary based on the network type and metric. For example, adult females showed the greatest decrease in affiliative closeness values across context, indicating that—with regard to interactions—they become the least connected group members along the road. Similar patterns have been seen in females’ risk-averse response to changes in captive environments^80^, and thus may be a typical network outcome in risky contexts. Conversely, adult females show some of the smallest decreases in proximity closeness and betweenness values across context. These results support the notion that females are more socially integrated into macaque groups^28^ and suggest that their risk-averse behaviour allows them to remain spatially connected with their associates. To our knowledge, this is the first study to demonstrate how proximity to humans has varied social network effects for different age-sex classes. Furthermore, the use of multiple network metrics underscores that there are different routes through which individuals can become more or less integrated into the group^63^. These shifts in social roles across context indicates flexibility in macaque social roles, which may be a key factor influencing macaque ecological flexibility in anthropogenic habitats; similar flexibility in social roles has been observed in pigtailed and rhesus macaques^31,83^.

Overall, we found support for life history theory predictions of how primates will incur risk by interacting with humans, but we demonstrate that social network factors influence these outcomes in nuanced ways. From a conservation perspective, females’ risk-aversion (adult females, in particular) may lower the risk of vulnerable individuals—i.e., infants and young juveniles^22,23^—being hit by passing vehicles. However, as juveniles become more independent they will lose any protection afforded by adult females’ risk-averse tendencies. Furthermore, males’ tendency to be risk-prone will likely facilitate continued frequent proximity to humans. We find that being in proximity to humans and/or provisioning opportunities disrupt macaque social networks, which is especially concerning given affiliative interactions and social bonds in primates are known to provide a suite of fitness benefits, including support from group members during agonistic interactions, increased social ranking, increased reproductive success, increased longevity, increased infant survival, and enhanced thermoregulation during winter^76,77,81^. As such, there are potentially direct fitness implications of such social network disruptions.

This study builds on our currently limited understanding of the relationship between social network position, life history variables, and primate behaviour in anthropogenic contexts. Future research on additional measures of risk perception, the extent to which dominance rank influence network position in tolerant macaque species, and the joint influence of forest food resource availability on tendency to interact with humans is needed to more fully understand what drives patterns of human-wildlife interaction in changing environments.

## Data Availability

Data used in this study are available upon request from the corresponding and senior author.

## References

[1] Wong BBM, Candolin U (2015). Behavioral responses to changing environments. Behav. Ecol..

[2] Purvis A, Gittleman JL, Cowlishaw G, Mace GM (2000). Predicting extinction risk in declining species. P. Roy. Soc. B- Biol. Sci..

[3] Knight J (2009). Making wildlife viewable: habituation and attraction. Soc. Anim..

[4] Chilvers BL, Corkeron PJ (2001). Trawling and bottlenose dolphins’ social structure. P. Roy. Soc. B- Biol. Sci..

[5] Fuentes A, O’Neill N, Shaw E, Cortés J (2007). Humans, monkeys, and the rock: the anthropogenic ecology of the Barbary macaques in the Upper Rock Nature Reserve, Gibraltar. Almoraima: revista de estudios Campo Gibraltareños..

[6] Guinn JE (2013). Generational habituation and current bald eagle populations. Hum-Wildl. Interact..

[7] Ziegltrum GJ (2008). Impacts of the black bear supplemental feeding program on ecology in western Washigton. Hum-Wildl Interact..

[8] Beisner BA (2015). Human-wildlife conflict: proximate predictors of aggression between humans and rhesus macaques in India. Am. J. Phys. Anthropol..

[9] Hsu MJ, Kao C-C, Agoramoorthy G (2009). Interactions between visitors and Formosan macaques (Macaca cyclopis) at Shou-Shan Nature Park, Taiwan. Am. J. Primatol..

[10] Fa, J. E. *Use of time and resources by provisioned troops of monkeys*. (Karger, 1986).

[11] Okamoto K, Matsumura S, Watanabe K (2000). Life history and demography of wild moor macaques (*Macaca maurus*): Summary of ten years of observations. Am. J. Primatol..

[12] Saj T, Sicotte P, Paterson JD (1999). Influence of human food consumption on the time budget of vervets. Int. J. Primatol..

[13] Burgin S, Hardiman N (2015). Effects of non-consumptive wildlife-oriented tourism on marine species and prospects for their sustainable management. J. Environ. Manage..

[14] Murray MH, Becker DJ, Hall RJ, Hernandez SM (2016). Wildlife health and supplemental feeding: a review and management recommendations. Biol. Cons..

[15] Fuentes A, Gamerl S (2005). Disproportionate participation by age/sex classes in aggressive interactions between long-tailed macaques (*Macaca fascicularis*) and human tourists at Padangtegal monkey forest, Bali, Indonesia. Am. J. Primatol..

[16] Jones-Engel L (2005). Primate-to-human retroviral transmission in Asia. Emerg. Infect. Dis..

[17] Chiyo PI, Alberts SC (2012). The influence of life history milestones and association networks on crop-raiding behavior in male African elephants. PloS One.

[18] McCarthy MS (2009). Sequences of Tibetan macaque (*Macaca thibetana*) and tourist behaviors at Mt. Huangshan, China. Primate Conserv..

[19] Sabbatini G, Stammati M, Tavares MCH, Giuliani MV, Visalberghi E (2006). Interactions between humans and capuchin monkeys (*Cebus libidinosus*) in the Parque Nacional de Brasília, Brazil. *Appl. Anim. Behav*. Science.

[20] Janson, C. H. & van Schaik, C. P. Ecological risk aversion in juvenile primates: Slow and steady wins the race. In *Juvenile Primates: Life History, Development, and Behavior*. (eds. Pereira, M. E. & Fairbanks, L. A.) 57–74 (University of Chicago Press, 2002).

[21] Wolf M, Doorn GS, van Leimar O, Weissing FJ (2007). Life-history trade-offs favour the evolution of animal personalities. Nature.

[22] Dunbar, R. I. M. Demography and reproduction. In *Primate Societies*. (eds. Smuts, B. B. & Bearder, S. K.), 240–249 (University of Chicago Press, 1987).

[23] Promislow DE, Harvey PH (1990). Living fast and dying young: A comparative analysis of life-history variation among mammals. J. Zool..

[24] Hockings KJ, Anderson JR, Matsuzawa T (2012). Socioecological adaptations by chimpanzees, *Pan troglodytes verus*, inhabiting an anthropogenically impacted habitat. Anim. Behav..

[25] Cantor M (2012). Disentangling social networks from spatiotemporal dynamics: the temporal structure of a dolphin society. Anim. Behav..

[26] Bode NWF, Wood AJ, Franks DW (2011). The impact of social networks on animal collective motion. Anim. Behav..

[27] Jacobs A, Sueur C, Deneubourg JL, Petit O (2011). Social network influences decision making during collective movements in brown lemurs (*Eulemur fulvus fulvus*). Int. J. Primatol..

[28] Fratellone GP (2019). Social connectivity among female Tibetan macaques (*Macaca thibetana*) increases the speed of collective movements. Primates.

[29] Croft DP (2005). Assortative interactions and social networks in fish. Oecologia.

[30] Krause J, Croft DP, James R (2007). Social network theory in the behavioural sciences: potential applications. Behav. Ecol. Sociobiol..

[31] Flack JC, Girvan M, De Waal FB, Krakauer DC (2006). Policing stabilizes construction of social niches in primates. Nature.

[32] Puga-Gonzalez I, Sosa S, Sueur C (2019). Editorial: Social networks analyses in primates, a multilevel perspective. Primates.

[33] Carne C, Semple S, MacLarnon A, Majolo B, Maréchal L (2017). Implications of Tourist–Macaque Interactions for Disease Transmission. EcoHealth.

[34] Tiddi B, Pfoh R, Agostini I (2019). The impact of food provisioning on parasite infection in wild black capuchin monkeys: a network approach. Primates.

[35] Blaszczyk MB (2017). Consistency in social network position over changing environments in a seasonally breeding primate. Behav. Ecol. Sociobiol..

[36] Kulahci IG, Ghazanfar AA, Rubenstein DI (2018). Knowledgeable Lemurs Become More Central in Social Networks. Curr. Biol..

[37] Supriatna, J., Shekelle, M. & Burton, J. *Macaca maura. The IUCN Red List of Threatened Species eT12553A3356200*; 10.2305/IUCN.UK.2008.RLTS.T12553A3356200.en (2008).

[38] Zak AA, Riley EP (2017). Comparing the use of camera traps and farmer reports to study crop feeding behavior of moor macaques (*Macaca maura*). Int. J. Primatol..

[39] Asquith PJ (1989). Provisioning and the study of free-ranging primates: History, effects, and prospects. Amer. J. Phys. Anthropol..

[40] Marty PR (2019). Time constraints imposed by anthropogenic environments alter social behaviour in longtailed macaques. Anim. Behav..

[41] Watanabe, K. & Matsumura, S. Social organization of moor macaques (*Macaca mauru*s) in the Karaenta Nature Reserve, South Sulawesi, Indonesia. In *Variations in the Asian Macaques* 147–162 (Tokai University Press, 1996).

[42] Matsumura S (1998). Relaxed dominance relations among female moor macaques (*Macaca maurus*) in their natural habitat, South Sulawesi, Indonesia. Folia Primatol..

[43] Riley EP, Sagnotti C, Carosi M, Oka NP (2014). Socially tolerant relationships among wild male moor macaques (*Macaca maura*). Behaviour.

[44] Supriatna J, Froehlich JW, Erwin JM, Southwick CH (1992). Population, habitat, and conservation status of *Macaca maurus, Macaca tonkeana* and their putative hybrids. Tropical Biodiversity.

[45] Bercovitch, F. B. Behavioral ecology and socioendocrinology of reproductive maturation in cercopithecine monkeys. In *Old World monkeys*. (eds. Whitehead, P. F. & Jolly, C. J.) 298–320 (Cambridge University Press, 2000).

[46] Altmann J, Alberts SC (2003). Variability in reproductive success viewed from a life-history perspective in baboons. Am. J. Hum. Biol..

[47] Coussi-Korbel S, Fragaszy DM (1995). On the relation between social dynamics and social learning. Anim. Behav..

[48] Thierry B (2000). The social repertoire of Sulawesi macaques. Primate Res..

[49] Castles M (2014). Social networks created with different techniques are not comparable. Anim. Behav..

[50] Croft, D. P., James, R. & Krause, J. *Exploring Animal Social Networks*. (Princeton University Press, 2008).

[51] Farine DR, Whitehead H (2015). Constructing, conducting and interpreting animal social network analysis. J Anim Ecol.

[52] James R, Croft DP, Krause J (2009). Potential banana skins in animal social network analysis. Behav. Ecol. & Sociobiol..

[53] Cairns SJ, Schwager SJ (1987). A comparison of association indices. Anim. Behav..

[54] Sundaresan SR, Fischhoff IR, Dushoff J, Rubenstein DI (2007). Network metrics reveal differences in social organization between two fission-fusion species, Grevy’s zebra and onager. Oecologia.

[55] Davis GH, Crofoot MC, Farine DR (2018). Estimating the robustness and uncertainty of animal social networks using different observational methods. Anim. Behav..

[56] McCarthy MS (2019). Camera traps provide a robust alternative to direct observations for constructing social networks of wild chimpanzees. Anim. Behav..

[57] Wheeler, R. E. multResp() *lmPerm*. *The R project for statistical computing*, http://www.r-project.org/ (2010).

[58] Sosa S (2016). The influence of gender, age, matriline, and hierarchical rank on individual social position, role and interactional patterns in *Macaca Sylvanus* at ‘La Forêt de Singes’: a multilevel social network approach. Front. Psychol..

[59] Matsumura S, Okamoto K (1997). Factors affecting proximity among members of a wild group of moor macaques during feeding, moving, and resting. Int. J. Primatol..

[60] Bonacich P (2007). Some unique properties of eigenvector centrality. Social Networks..

[61] Kawazoe T, Sosa S (2019). Social networks predict immigration success in wild Japanese macaques. Primates.

[62] Bercovitch, F. B. & Harvey, N. C. Reproductive life history. In *Macaque Societies: A Model for the Study of Social Organization*. (eds. Thierry, B., Singh, M. & Kaumanns, W.) 41, 61–83 (Cambridge University Press, 2004).

[63] Thierry, B., Singh, M. & Kaumanns, W. *Macaque Societies: A Model for the Study of Social Organization*. **41**, (Cambridge University Press, 2004).

[64] Handcock, M. S., Hunter, D. R., Butts, C. T., Goodreau, S. M. & Morros, M. statnet: Software tools for the statistical modeling of network data, http://statnetproject.org (2003). 10.18637/jss.v024.i01 PMC2447931 18618019

[65] Butts, C. T. network: A package for managing relational data in R, https://cran.r-project.org/web/packages/network/vignettes/networkVignette.pdf (2015).

[66] Butts, C. T. sna: Tools for social network analysis, http://www.statnet.org (2016).

[67] Csardi, G. & Nepusz, T. The igraph software package for complex network research, http://igraph.org (2006).

[68] Neuwirth, E. RColorBrewer: ColorBrewer palettes, https://CRAN.R-project.org/package=RColorBrewer (2013).

[69] Beisner, B. A., Jackson, M. E., Cameron, A. N. & McCowan, B. Detecting instability in animal social networks: Genetic fragmentation is associated with social instability in rhesus macaques. *PloS one* **6**, e16365; 10.1371/journal.pone.0016365 (2011). 10.1371/journal.pone.0016365 PMC3027651 21298105

[70] R Core Team. R: A language and environment for statistical computing, https://www.R-project.org/ (2019).

[71] Yang Z, Algesheimer R, Tessone CJ (2016). A comparative analysis of community detection algorithms on artificial networks. Scientific reports.

[72] Higham JES, Shelton EJ (2011). Tourism and wildlife habituation: Reduced population fitness or cessation of impact?. Tourism Management.

[73] Cibot M, Bortolamiol S, Seguya A, Krief S (2015). Chimpanzees facing a dangerous situation: A high-traffic asphalted road in the Sebitoli area of Kibale National Park, Uganda. Amer. J. Primatol..

[74] Hockings K, James R (2006). Anderson & Tetsuro Matsuzawa. Road crossing in chimps: a risky business. Curr. Biol..

[75] Henzi SP, Barrett L (2007). Coexistence in female-bonded primate groupjs. Adv. Stud. Behav..

[76] Silk JB (2007). Social components of fitness in primate groups. Science.

[77] Silk JB (2010). Strong and consistent social bonds enhance the longevity of female baboons. Curr. Biol..

[78] Kasper C, Voelkl B (2009). A social network analysis of primate groups. Primates.

[79] Puga-Gonzalez I, Sosa S, Sueur C (2019). Social style and resilience of macaques’ networks, a theoretical investigation. Primates.

[80] Koyama NF, Aureli F (2019). Social network changes during space restriction in zoo chimpanzees. Primates.

[81] McFarland R (2015). Social integration confers thermal benefits in a gregarious primate. J. Anim. Ecol..

[82] Wrangham, R. Evolution of social structure. In *Primate Societies*. (eds. Smuts, B. B. & Bearder, S. K.) 282–296 (The University of Chicago Press, 1987).

[83] Liao Z, Sosa S, Wu C, Zhang P (2017). The influence of age on wild rhesus macaques’ affiliative social interactions. Amer. J. Primatol..

[84] Wasserman, S. & Faust, K. *Social network analysis: Methods and applications*. 8 (Cambridge university press, 1994).

